# A Novel Method of Early Diagnosis of Alzheimer's Disease Based on EEG Signals

**DOI:** 10.1155/2015/931387

**Published:** 2015-01-19

**Authors:** Dhiya Al-Jumeily, Shamaila Iram, Francois-Benois Vialatte, Paul Fergus, Abir Hussain

**Affiliations:** ^1^Applied Computing Research Group, Liverpool John Moores University, Byrom Street, Liverpool L3 3AF, UK; ^2^Laboratoire SIGMA, ESPCI ParisTech, 14 boulevard des Frères Voisin, 92130 Issy-les-Moulineaux, France

## Abstract

Studies have reported that electroencephalogram signals in Alzheimer's disease patients usually have less synchronization than those of healthy subjects. Changes in electroencephalogram signals start at early stage but, clinically, these changes are not easily detected. To detect this perturbation, three neural synchrony measurement techniques: phase synchrony, magnitude squared coherence, and cross correlation are applied to three different databases of mild Alzheimer's disease patients and healthy subjects. We have compared the right and left temporal lobes of the brain with the rest of the brain areas (frontal, central, and occipital) as temporal regions are relatively the first ones to be affected by Alzheimer's disease. Moreover, electroencephalogram signals are further classified into five different frequency bands (delta, theta, alpha beta, and gamma) because each frequency band has its own physiological significance in terms of signal evaluation. A new approach using principal component analysis before applying neural synchrony measurement techniques has been presented and compared with Average technique. The simulation results indicated that applying principal component analysis before synchrony measurement techniques shows significantly better results as compared to the lateral one. At the end, all the aforementioned techniques are assessed by a statistical test (Mann-Whitney *U* test) to compare the results.

## 1. Introduction

Mild cognitive impairment (MCI) is characterized by impaired memory state of brain probably leading towards mild Alzheimer's disease (MiAD) or Alzheimer's disease (AD). This prodromal stage of AD has been under a great influence of research for a long time [1–3]. Statistics reported that 6–25% of MCI is transformed to AD annually and 0.2–4% from healthy person to AD [2, 4], revealing the fact that MCI is a transition state of MiAD and AD.

Loss of functional connectivity between cortical and hippocampus has long been an important focus of researches to examine the cause of cognitive dysfunction in AD [5, 6]. Statistical analysis of interdependence among time series recorded from different brain areas, to study the functional interaction, is called “functional connectivity” [7]. Due to destructive characteristics of AD, it has also been characterized as a neocortical “disconnection syndrome” [8]. The brain's visualization as a complex network of subsystems has led us to find out the factors that can best identify functional disorders in brain [9]. There is now ample evidence that formation of dynamic links in terms of synchronization constitutes the functional integration of the brain [10–12].

Electroencephalogram (EEG) signals are considered functional examples to evaluate cognitive disturbances and a diagnostic tool, especially when a diagnostic doubt exists even after the initial clinical procedures [13, 14]. A great deal of research has already been conducted to detect the fluctuations in EEG signals [2, 5, 15]. Alteration in the regional cerebral blood flow (rCBF) has been considered one of the causes of abnormality in EEG signals of AD [16, 17]. Studies on MCI have shown a decrease of alpha power [18, 19] and an increase of theta (4–8 Hz) power [20, 21] in corticocortical and subcortical parts of the brain. Babiloni et al. [2] claimed that the reduction of the synchronization likelihood occurs both at interhemispherical (delta-beta2) and frontoparietal (delta-gamma) electrodes.

Topographically analyzing the EEG signals, Hogan et al. [22] reported a less synchronization of upper alpha band between central and temporal cortices. In line, a correlation between higher low-frequency amplitude and alpha-beta activity at frontal region may reflect an early sign of cortical atrophy during the course of AD [23]. Similarly, perturbation in cholinergic inputs from the basal forebrain to cortex and hippocampus indicates a decrease in cortical EEG coherence [24] that can be considered a biomarker for the early detection of AD [2]. Moreover, a combination of multilinear interactions within the tensor formed by multiplying the* subject *×* frequency *×* regions* also provides a simple set of features for the interpretation and classification of AD at its early stage [25]. The concept of* local* and* global* methods is used to analyze synchronization between pairs of signals and entire EEG channels at the same time, respectively [15].

The studies, so far, have provided a very limited regional comparison of brain; for instance, less synchronization has been reported between temporal and central regions [22] and also in frontoparietal region [2]. Similarly, functional coupling of EEG rhythms by sensorimotor events is presented only in centroparietal regions of brain [26]. A wider range of study is still required to analyze the synchronization likelihood in all parts of brain (right temporal, left temporal, frontal, central, and occipital) at the same time, on different sets of data for AD.

Synchronization, precisely speaking, is a coordination of “rhythmic oscillators” [27] for a repetitive functional activity, whereas neural synchronization is putatively considered a mechanism where brain regions simultaneously communicate with each other to complete a specific task such as perception, cognition, and action [28, 29]. Any disturbance in the brain, caused by a disease or any other infection, can highly affect the synchronization of brain. Quantitative analysis of EEG signals provides a better insight of synchronization between different parts of brain. For instance, less synchrony has been detected in the EEG signals of AD patients as compared to healthy persons [15].

Various synchrony measurement techniques have already been discussed to detect any perturbation in the EEG signals of AD patients [30]. Both linear such as coherence and nonlinear such as phase synchronization methods are widely used to quantify synchronization in electroencephalographic signals [6, 31, 32]. A comparison of occipital interhemispheric coherence (IHCoh) for normal older adults and AD patients reveals a reduced occipital IHCoh for both lower and higher bands of alpha [33]. Almost similar findings were reported by Locatelli et al. [34] where a significant increase in delta coherence is noticed between frontal and posterior regions in AD patients while a decrease in alpha coherence is shown in temporoparietooccipital areas. Spontaneous phase synchronization of different brain regions is calculated by Kuramoto's parameter (*ρ*), which is particularly useful to measure multichannel data [6].

Despite the considerable success of the above mentioned techniques to analyze disruption in the EEG signals of Alzheimer's patients, further investigations are still required to fulfill the clinical requirements. For instance, in order to detect Alzheimer's disease at its earlier stages, we need to focus on those areas where Alzheimer's disease attacks at first and then we need to check its synchronization with the rest of the brain regions. Furthermore, additional novel and comprehensive methods are still required to check the validity of aforementioned techniques on EEG signals.

The above overview suggests that, first, spatial-spectral analysis of EEG signals can provide a measure of memory visualization. Second, neural synchrony measurement techniques have a potential to discriminate between AD patients and healthy subjects. What is still missing or ambiguous in the literature survey is the simultaneous comparison of all parts of brain with the right and left temporal lobes (the most affected parts of brain) to analyze synchronization and also the implementation of new methods to apply synchrony measurement techniques. In this research work, the following novel contributions are considered.

- We have filtered a dataset of MiAD patients into five different frequency bands (delta, theta, alpha, beta, and gamma). For each frequency band, we have computed neural synchronization to compare all parts of brain (frontal, occipital, and central) with left and right temporal lobes.
- Furthermore, three different sets of MiAD patients are compared to check the validity of our methodology. A high intersubject variability has been seen in the EEG signals of AD patients, especially with different level of severity and comorbidities [25, 35, 36]. Most of the existing studies focus on a single synchrony measure with a single set of data [37]. Also, they apply different measures to different datasets. In this case, it is hard to compare the results to conclude a single hypothesis. To extract a general set of features, we have analysed three different databases, each from one hospital at a time.
- In order to remove the ambiguity of biased results due to “features redundancy,” we have applied PCA (principal component analysis) technique before applying synchrony measurement techniques. Reducing features vector dimension, commonly known as feature reduction, will help to get accuracy results and avoid overfitting classification [38]. We compare the results with simple Average technique to analyze the pros and cons of the new proposed methodology.

Besthorn et al. [39] applied PCA technique in the quantitative analysis of EEG signals to compress a group of predictor variables to a small set of factors or principle components. Later, they applied linear discriminant classifier to these variables to discriminate AD patients from healthy subjects. Similarly, Uhlhaas et al. [40] applied PCA to remove the artifacts from EEG signals that were generated by eye-blink. To the best of our knowledge and the literature we have surveyed so far, we could not find the application of PCA to remove the redundant features from the data that can generate a biased result to check the synchronization of brain areas.

Given the exploratory nature of the study, our priori hypothesis is that the proposed methodology would provide a better insight to investigate the decline in the neural synchronization of AD patients. It would provide a better topographical and spectral analysis of the brain regions eliminating the probability of biased result due to feature redundancy.

The rest of this paper is structured as follows.  Section 2 provides an overview of our synchrony measurement techniques, the utilized data and the filtering process using five frequency bands, methodology of the proposed technique, and statistical analysis of the results. Sections 3 and 4 are dedicated to discussion and conclusion, respectively.

## 2. Methods

### 2.1. Synchrony Measurement Techniques

**Figure 1:**
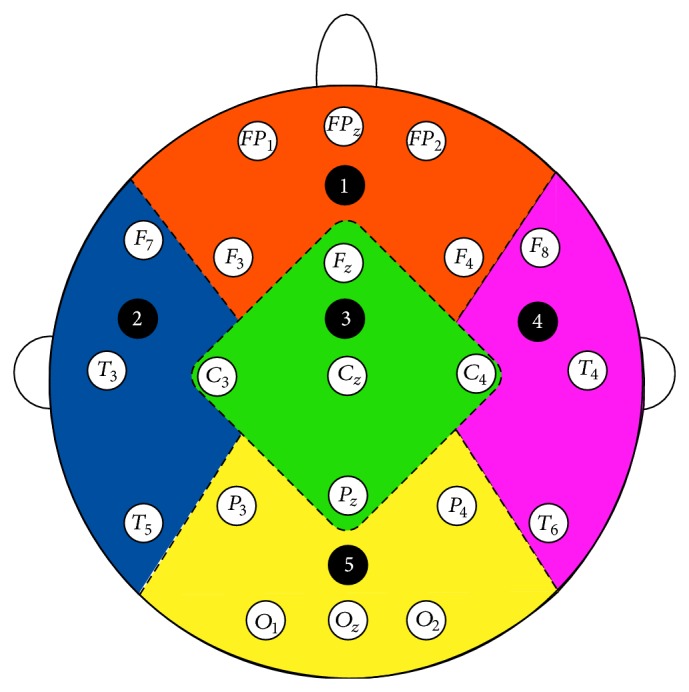
The 21 channels used for EEG recording [15].

In this section, we briefly review the synchrony measurement techniques that we have implemented in our datasets which include phase synchrony, cross correlation, and coherence. For this research work, we have selected three synchrony measures from the literature that provides comparatively better results when implemented in EEG signals for the diagnosis of Alzheimer's disease [15]. We use these three synchrony measures to infer which of our proposed methods provides better results in terms of *P* values.  Figure 1 shows the 21 channels used for EEG recording.

#### 2.1.1. Phase Synchrony (Hilbert Transform)

The oscillation of two or more cyclic signals where they tend to keep a repeating sequence of relative phase angles is called phase synchronization. Synchronization of two periodic nonidentical oscillators refers to the adjustment of their rhythmicity, that is, the phase locking between the two signals [41, 42]. It refers to the interdependence between the instantaneous phases *φ*_1_(*t*) and *φ*_2_(*t*) of the two signals *s*_1_(*t*) and *s*_2_(*t*), respectively. It is usually written as

(1) $$\begin{array}{c} \varphi_{m,n}=m\varphi_{1}\left(t\right)-n\varphi_{2}\left(t\right)=\text{constant}, \end{array}$$

where *m* and *n* are integers indicating the ratio of possible frequency locking and *φ*_*m*,*n*_ is their relative phase or phase difference. To compute the phase synchronization, the instantaneous phase of the two signals should be known. This can be detected using analytical signals based on Hilbert transform [9] as follows:

(2) $$\begin{array}{c} z\left(t\right)=x\left(t\right)+i\tilde{x}\left(t\right). \end{array}$$

Here, *z*(*t*) is complex value with *x*(*t*) being a real time series and $\tilde{x}(t)$ being its Hilbert transform. The Hilbert transform can be calculated as

(3) $$\begin{array}{c} \tilde{x}\left(t\right)=\frac{1}{\pi }\text{PV}\overset{\infty }{\underset{-\infty }{\int }}\frac{x\left(\tau \right)}{t-\tau }dt. \end{array}$$

Here, PV denotes Cauchy principle value. The instantaneous phases *φ*_1_(*t*) and *φ*_2_(*t*) for both signals can be calculated with the following formula:

(4) $$\begin{array}{c} \varphi \left(t\right)-\arctan\frac{\tilde{x}\left(t\right)}{x\left(t\right)}. \end{array}$$

#### 2.1.2. Cross Correlation

Cross correlation is a mathematical operation used to measure the extent of similarity between two signals. If a signal is correlated to itself, it is called autocorrelated. If we suppose that *x*(*n*) and *y*(*n*) (why not *s*_1_(*t*) and *s*_2_(*t*) make uniform signals suggestion) are two time series, then the correlation between them is calculated as [43]

(5) $$\begin{array}{c} {\hat{R}}_{xy}\left(m\right)=\{\begin{array}{cc} \overset{N-m-1}{\underset{n=0}{\sum }}x_{n+m}y_{n} & m\geq 0\\ {\hat{R}}_{yx}\left(-m\right) & m<0. \end{array} \end{array}$$

Cross correlation returns a sequence of length 2∗*N* − 1 vector, where *x* and *y* are of length *N* vectors (*N* > 1). If *x* and *y* are not of the same length, then the shorter vector is zero-padded. Cross correlation returns value between −1 and +1. If both signals are identical to each other, the value will be 1; if they are totally different from each other, then the cross correlation coefficient is 0, and if they are identical with the phase shift of 180°, then the cross correlation coefficient will be −1 [15].

#### 2.1.3. Magnitude Squared Coherence

The coherence functions estimate the linear correlation of signals in frequency domain [15]. The magnitude squared coherence is defined as the square of the modulus of the mean cross power spectral density (PSD) normalized to the product of the mean auto PSDs [44]. The coherence *C*_*xy*_(*f*) between two channel time series is computed as

(6) $$\begin{array}{c} C_{xy}\left(f\right)=\frac{\left|P_{xy}\left(f\right)\right|}{P_{xx}\left(f\right)P_{yy}\left(f\right)}, \end{array}$$

where *P*_*xy*_(*f*) is the cross PSD estimate of *x* and *y* and *P*_*xx*_(*f*) and *P*_*yy*_(*f*) are the PSD estimates of *x* and *y*, respectively.

For discrete signals *x* and *y*, cross power spectral densities (*P*_*xy*_(*f*)) can be calculated with the given formula as follows:

(7) $$\begin{array}{c} P_{xy}\left(f\right)=\underset{T\to \infty }{\lim}E\left\{{\left[F_{x}^{T}\left(w\right)\right]}^{*}F_{y}^{T}\left(w\right)\right\}. \end{array}$$

Here, cross spectral density, which is also known as cross power spectrum, is the Fourier transform of the cross correlation function

(8) Pxy(f)=∫−∞∞Rxy(t)e−jwtdt=∫−∞∞·[∫−∞∞(x(t))·y(T+t)dT]e−jwtdt,

where *R*_*xy*_(*t*) is the cross correlation of *x*(*t*) and *y*(*t*). On the other side, autopower spectral densities (*P*_*xx*_(*f*) and *P*_*yy*_(*f*)) for *x*(*t*) and *y*(*t*) can be calculated from the autocorrelation instead of cross correlation functions. Consider

(9) $$\begin{array}{c} P_{xx}\left(f\right)=\underset{T\to \infty }{\lim}E\left\{{\left[F_{x}^{T}\left(w\right)\right]}^{*}F_{x}^{T}\left(w\right)\right\},\\ P_{yy}\left(f\right)=\underset{T\to \infty }{\lim}E\left\{{\left[F_{y}^{T}\left(w\right)\right]}^{*}F_{y}^{T}\left(w\right)\right\}. \end{array}$$

### 2.2. Data Description and Data Filtering

#### 2.2.1. Data Description

The datasets that we are analyzing have been recorded from three different countries of European Union. Specialist at the memory clinic referred all patients to the EEG department of the hospital. All patients passed through a number of recommended tests: minimental state examination (MMSE) [45], the Rey Auditory Verbal Learning Test [46], Benton Visual Retention test [47], and memory recall tests [48]. The results are scored and interpreted by psychologists and a multidisciplinary team in the clinic. After that, each patient is referred to hospital for EEG assessment to diagnose the symptoms of AD. Patients were advised to be in a resting state with their eyes closed during the test. The sampling frequency and number of electrodes for three datasets are all different. Detailed information is as follows.

#### 2.2.2. Database A

The EEG* dataset A* contains 17 MiAD patients (10 males; aged 69.4 ± 11.5 years) and 24 healthy subjects (9 males; aged 77.6 ± 10 years). They all are of British nationality. This data was obtained using a strict protocol from Derriford Hospital, Plymouth, UK, and has been collected using normal hospital practices. EEG signals were obtained using the modified Maudsley system which is similar to the traditional 10–20 international system [49]. EEGs were recorded for 20 sec at a sampling frequency of 256 Hz (later on sampled down to 128 Hz) using 21 electrodes.

#### 2.2.3. Database B

This EEG dataset is composed of 5 MiAD patients (2 males; aged 78.8 ± 5.6 years) as well as 5 healthy subjects (3 males; aged 76.6 ± 10.0 years). They all are of Italian nationality. Several tests, for instance, MMSE, the clinical dementia rating scale (CDRS), and the geriatric depression scale (GDS), were conducted to evaluate the cognitive state of the patients. The MMSE result for healthy subjects is 29.3 ± 0.7, while for MiAD patients is 22.3 ± 3.1. EEGs were recorded for 20 sec at a sampling frequency of 128 Hz using 19 electrodes at the University of Malta, Msida MSD06, Malta.

#### 2.2.4. Database C

This dataset consists of 8 MiAD patients (6 males; aged 75 ± 3.4 years) and 3 healthy subjects (3 males; aged 73.5 ± 2.2 years). They all are of Romanian nationality. The AD patients have been referred by a neurologist for EEG recordings. All subjects are diagnosed with AD by means of psychometric tests (MMSE, CDR, and OTS), neuroimaging (CT), and clinical examination (gender, age, disease, duration, education, and medication). The MMSE result for healthy subjects is 28–30, while for MiAD patients it is 20–25. EEG data is recorded using a large equidistant 22-channel arrangement conforming to the international federation of clinical neurophysiology (IFCN) standards [50] for digital recording of clinical EEG from the Ecological University of Bucharest. The time series are recorded for 10 to 20 minutes at a sampling frequency of 512 Hz using 22 electrodes. The signals are notch-filtered at 50 Hz. Further details about the data can be found in [51].

For current research work, we have obtained a version of the data that is already preprocessed of artifacts by using independent component analysis (ICA), a blind source separation technique (BSS). Details of these procedures can be found in [52]. For ICA processed data, the least corrupted 20 s recordings have been selected for further analysis.

#### 2.2.5. Data Filtering into Five Frequency Bands

EEG time series are classified into five frequency bands. Each frequency band has its own physiological significance [6, 53] as follows.

- Delta (*δ*: 1 ≤ *f* ≤ 4 Hz): these are characterized by deep sleep and are correlated with different pathologies.
- Theta (*θ*: 4 ≤ *f* ≤ 8 Hz): these play an important role during childhood. High theta activities in adults are considered abnormal and associated with brain disorders.
- Alpha (*α*: 8 ≤ *f* ≤ 12 Hz): these usually appear during mental inactive conditions and under relaxation. They are best seen during closing of eye and mostly pronounced in occipital location.
- Beta (*β*: 12 ≤ *f* ≤ 25 Hz): these are visible in central and frontal locations. Their amplitude is less than alpha waves and they are mostly enhanced during tension.
- Gamma (*γ*: 25 ≤ *f* ≤ 30 Hz): these are best characterized by cognitive and motor functions.

Bandpass filter is applied to each EEG channel to extract the EEG data in specific frequency band [*F*: (*F* + *W*)] Hz. Butterworth filters were used (of 2nd order) as they offer good transition band characteristics at low coefficient orders; thus, they can be implemented efficiently [54].

### 2.3. Methodology

In this research work, a novel methodology using PCA and neural synchrony measurement of the brain is proposed. We have compared our proposed method with other methods which takes the average of synchrony measures for all channels in one region of the brain. As mentioned previously, we are comparing the right and left temporal lobes with the frontal, central, and occipital areas; so, there are a total of 7 comparisons of the brain ((left temporal-right temporal (LT-RT)), (left temporal-frontal (LT-F)), (left temporal-central (LT-C)), (left temporal-occipital (LT-O)), (right temporal-frontal (RT-F)), (right temporal-central (RT-C)), and (right temporal-occipital (RT-O))) for all frequency bands (*δ*, *θ*, *α*, *β*, and *γ*). A brief description of these methods is given below.

#### 2.3.1. First Method (Taking Average of Synchrony Measures for All Channels of One Region)

First, we apply neural synchrony measurement techniques to each channel pair (time series of two channels) of two different regions for all frequency bands and then we take the average of those results. For instance, we apply phase synchrony measure to each channel pair of right and left temporal lobes ((*F*_7_-*F*_8_), (*F*_7_-*T*_4_), (*F*_7_-*T*_6_), (*T*_3_-*F*_8_), (*T*_3_-*T*_4_), (*T*_3_-*T*_6_), (*T*_5_-*F*_8_), (*T*_5_-*T*_4_), and (*T*_5_-*T*_6_)) and then we take the average result of right temporal-left temporal. We compare the left temporal lobe with the frontal (*FP*_1_,* FP*_2_, *FP*_*z*_,* F*_3_, and* F*_4_), central (*F*_*z*_,* C*_3_, *C*_*z*_,* C*_4_, and *P*_*z*_), and occipital (*P*_3_,* P*_4_,* O*_1_,* O*_2_, and *O*_*z*_) areas. Similarly, we compare the right temporal lobe (*F*_8_,* T*_4_, and* T*_6_) to the rest of the brain area. The same technique has been used for the rest of the synchrony measures, that is, cross correlation and coherence.

**Figure 2:**
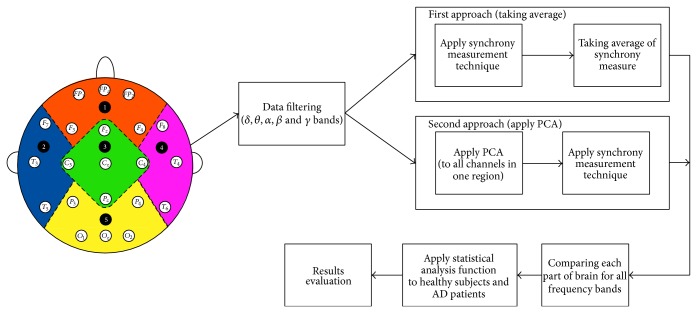
Average and PCA methods.

After getting the results, we compare the neural synchronization of AD patients and healthy subjects, for all three measurement techniques (phase synchronization, cross correlation, and coherence), by Mann-Whitney *U* test.  Figure 2 shows all the steps of our Average method.

#### 2.3.2. Second Method (PCA Based Neural Synchrony Measure)

In this method, instead of applying synchrony measurement techniques directly to the filtered data, first we apply principal component analysis (PCA) technique to all channels of one. This eliminates any redundant information that a region could provide. For instance, we apply PCA to all three channels of left temporal lobe (*F*_7_,* T*_3_, and* T*_5_) and consequently it provides a single signal without any redundant information. Then, we apply PCA to all channels of right temporal lobe (*F*_8_,* T*_4_, and* T*_6_). After that, we apply synchrony measure to these two regions. Similarly, we apply PCA to all other channels of a region: frontal (*FP*_1_,* FP*_2_, *FP*_*z*_,* F*_3_, and* F*_4_), central (*F*_*z*_,* C*_3_,* C*_*z*_,* C*_4_, and *P*_*z*_), and occipital (*P*_3_,* P*_4_,* O*_1_,* O*_2_, and* O*_*z*_) and compute the synchrony measure with left and right temporal lobes. The rest of the procedure is similar to the first proposed method.

#### 2.3.3. Principal Component Analysis (PCA)

The basic purpose of PCA is to reduce the dimensionality of a dataset to convert it to uncorrelated variables providing maximum information about a data while eliminating interrelated variables. In other words, it transforms the highly dimensional dataset (of *m* dimensions) into low dimensional orthogonal features (of *n* dimension) where *n* < *m* [55].

**Figure 3:**
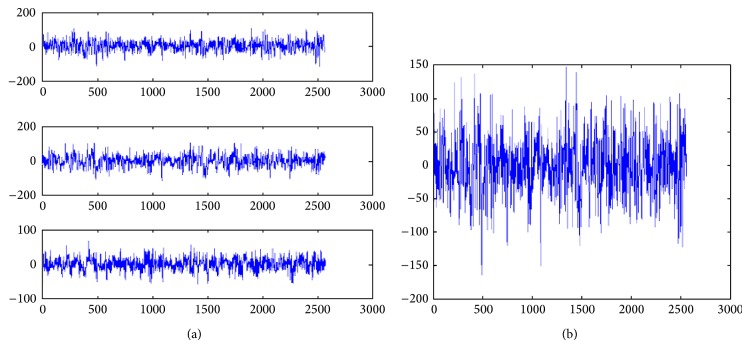
Application of PCA on left temporal lobe channels signals.

In our case, we apply PCA to all channels in one particular region, for instance, the application of PCA for the left temporal lobe as shown in Figure 3(a) using channel (*F*_7_,* T*_3_,* T*_5_) are converted into a single signal as shown in Figure 3(b). The generated temporal signal contains almost all information from the left temporal lobe while eliminating any redundant information.

### 2.4. Statistical Analysis

To investigate whether there is a significant difference between the EEG signals of MiAD patients and the control subjects and also to prove the probable significance of our proposed methodology, we apply the Wilcoxon rank sum (Mann-Whitney) test [56, 57] to our datasets. A rank sum function is a nonparametric test which allows us to check whether the statistics at hand, in our case synchrony results, take different values from two different populations. Lower *P* values indicate higher significance in terms of large difference in medians of two populations [15].

Since we are applying three different synchrony measures to three different sets of data, first we consider our first proposed method (taking average of synchrony values) to compute the synchrony measure. We apply all three measures for all 7 different comparisons of brain for all frequency bands and compute the results by Mann-Whitney test. Then, we apply the same techniques on all, above mentioned, three datasets using the second proposed method (PCA based synchrony measures). This will enable us to compare our results in two different perspectives as follows.

- Investigating three different synchrony measures at a time will help us to compare which measure works better for EEG signals.
- Secondly, we are able to compare two different methods for three synchrony measures using three different datasets.

In addition to evaluating the statistical significance of our proposed method, this will also help us to differentiate the MiAD patients from healthy subjects.

## 3. Results and Discussions

The aim of the present study is to find the relationship of EEG synchronization with AD and thus to explore further dimensions in disconnection theorem of cognitive dysfunction in AD and also to investigate a better method to detect any changes in EEG synchrony that can be considered a biomarker for the early detection of AD. Here, we investigate and discuss results in two different angles. First, we discuss the role of synchrony measures to examine a change in EEG synchrony in MiAD patients and later we confer the significance of applying PCA before synchrony measures.

### 3.1. Functional Disconnection of Brain Regions due to Lower Synchronization

We have observed that all of the synchrony measures, tested in this paper, show a decrease in EEG synchrony for MiAD patients as compared to healthy subjects. However, cross correlation shows a higher number of significant results at the *P* = 0.01 level as compared to phase synchrony and coherence. We have examined mostly the areas that have shown less functional connectivity for all three synchrony measures, which are; right temporal-central (RT-C) for delta, theta, and alpha bands and also left temporal-occipital (LT-O) for delta and alpha bands. The rest of this paper discusses these two regions where we find highly significant results compared to the rest of the regions.

First, we discuss* dataset A* for all three synchrony measures with PCA based method. The *P* values for cross correlation in RT-C region are 2.47 × 10^−4^, 1.46 × 10^−4^, and 0.009 for delta, theta, and alpha bands, respectively. In LT-O region, the smallest *P* values for delta and theta bands are 8.50 × 10^−5^ and 6.8 × 10^−5^, respectively. The 2nd best measure which has given us remarkable results is phase synchrony, where we get 0.0067, 0.0403, and 0.0585 *P* values for delta, theta, and alpha bands, respectively, in RT-C region. We get 0.0041 and 0.0271 *P* values for delta and alpha bands in LT-O region. Lastly, the coherence function shows significant results in RT-C region for delta band, *P* value = 0.0378, and in LT-O 9.8 × 10^−4^ and 0.05 for delta and alpha bands, respectively. Coherence function does not provide significant results and hence contradicts Bahar theory [58] where control group showed higher values of evoked coherence in delta, theta, and alpha bands in the left frontoparietal electrode pairs as compared to AD patients.

Lower *P* values at delta and alpha bands are shown by Babiloni et al. [2] at frontoparietal couplings of electrodes which indicates a lower synchronization in MCI and AD subjects. Further to the previous findings, our results show a higher difference of synchronization for temporal, occipital, and central areas in MiAD patients at delta, theta, and alpha levels. They show lower magnitudes of delta, theta, and alpha bands in temporal, central, and occipital areas in MiAD patients than the compared healthy subjects. Temporal regions are characterized by short term and long term memory and any neuronal change on these sites is a clear indication of progression of AD.

Interestingly, we find a decrease in alpha band synchronization for all three synchrony measures in almost all regions. For instance, for cross correlation, *P* value < 0.01 in almost all parts of the brain; for phase synchrony, the *P* values are 0.058, 0.0038, 0.011, and 0.027 in RT-C, RT-O, RT-F, and LT-O, respectively. This shows the importance of alpha rhythm for the early detection of AD which is in accordance with the phenomena that alpha rhythms are mainly modulated by thalamocortical and corticocortical systems [56, 57]. Alpha band is mainly related to subjects global attentional readiness and engagement of specific neural channels for the elaboration of sensorimotor or semantic information [2].

As aforementioned, mostly the areas that show lower dysfunctional connectivity are right temporal-central and left temporal-occipital. A lower synchronization in these connections, especially in RT-C region, for alpha band indicates a disturbance in the perception and integration of somatosensory information, visuospatial processing, and cognitive disorder. This information is in line with clinical findings presented in [59] for increasing visual and spatial deficits in MCI and MiAD patients.  Table 1 shows the significant *P* values in different parts of the brain in different frequency bands for* dataset A*.

Similarly, for* dataset B* and* dataset C*, we found low *P* values in the same regions for same frequency bands but not as much significant as for* dataset A*. One thing in common in all three datasets is that they show lower *P* values in alpha frequency bands in the RT-C region.  Table 2 shows the total number of significant values in case of PCA and Average method.

### 3.2. Significance of PCA Approach over Average Approach

Our second hypothesis was to show the significance of using PCA techniques to eliminate the redundant information from the data that can give biased results, before applying synchrony measures. As expected, we found a big difference in results with and without PCA method. We have found that more than 90% of the values are better in case of* PCA* method as compared to Average method for all of three datasets.

For instance, for* dataset A*, in case of PCA method, we have found 8 significant values below 0.01 (*P* < 0.01) and 11 significant values below 0.05 (*P* < 0.05), while only 2 values below 0.01 (*P* < 0.01) and 8 values below 0.05 (*P* < 0.05) in case of Average method for phase synchrony measure were found. Similarly, for cross correlation measures, although the difference is not very high, yet the PCA method has shown more significant values. For example, the number of *P* values below 0.01 (*P* < 0.01) is 26, while almost all 35 values were below 0.05 (*P* < 0.05); for Average method 22 values are below 0.01 and 30 values are below 0.05 (*P* < 0.05). As aforementioned, coherence function does not perform better as compared to other two synchrony measures but again we found more significant results in case of* PCA* method as compared to the Average method.

**Figure 4:**
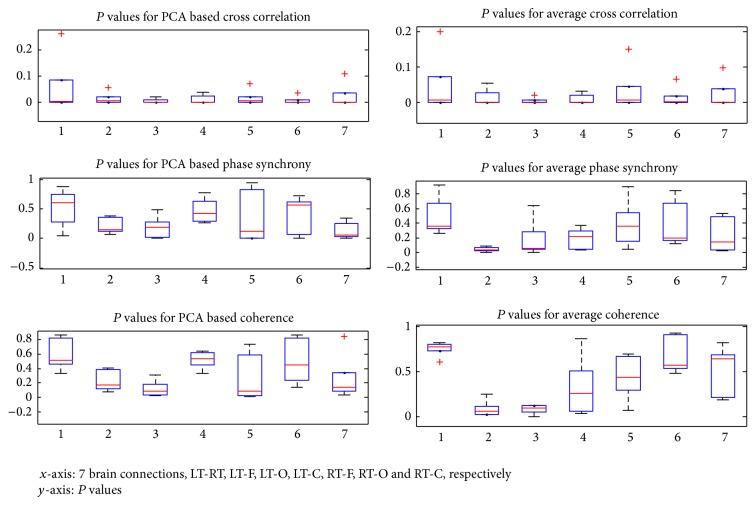
Boxplots show the results of three synchrony measures for PCA and Average methods.

The reults are also shown by boxplot in Figure 4 that show the difference of *P* values for all three synchrony measures in all 7 brain comparsions for* dataset A*. They compare the results of synchrony measures for PCA and Average methods.

Similarly, for* dataset B* and* dataset C*, the results of PCA method are more significant as compared to Average method. This clearly shows that using PCA method before synchrony measures has two advantages as follows.

- As the redundant information is eliminated from the datasets, the results are not biased and are more reliable.
- Secondly, it proves that application of PCA generates more significant results as compared to average synchrony measure method.

## 4. Conclusion

The aim of the current study was to show the significance of applying PCA method to eliminate redundant information from the datasets to get more reliable results. In this study, three different datasets were selected with different specifications and three different synchrony measures are applied to prove the significance of our approach. Moreover, we have compared our proposed method with Average methods to compute synchronization in MiAD patients as well as in control subjects.

Results revealed that cross correlation measure showed higher difference in synchronization of MiAD and control subjects as compared to phase synchrony, while coherence function did not perform very well. They have also indicated that alpha and theta bands play a major role in identifying the change in synchronization from MiAD and control subjects especially in right temporal-central region (RT-C) and also in left temporal-occipital (LT-O) region.

Furthermore, the original contribution of this research work is the comparison of previous methods of applying synchrony measures with PCA based method. Our proposed method proved the importance of eliminating redundant information, from EEG time series, that may come from consecutive electrodes. It should be noted that comparison with previous findings is problematic due to the significant differences in the utilized methodology and the utilization of different kinds of synchrony measures on different kinds of datasets. However, our results are consistent with most of the studies on the loss of average EEG synchrony in different parts of the brain for MiAD patients and are also in accordance with the clinical findings.

Furthermore, we have successfully shown the importance and significance of our proposed method, to detect lower synchronization in MiAD patients, as compared to the Average method for all three datasets.

Future work will involve the study of much significant results of lower synchronization in case of* dataset B* and* dataset C* as compared to* dataset A*. In this paper, we have implemented PCA to eliminate the redundant and irrelevant information from the EEG signals and also applied signal processing techniques to extract the features that are useful for the early diagnosis of Alzheimer's disease. In this ongoing research project, the next step is the implementation of classification algorithms to recognize the data patterns that can be used for the identification and diagnosis of Alzheimer's disease in clinics.

## Figures and Tables

**Table 1:** *P* values for *dataset A*, different frequency bands in different brain connections.

Synchrony measure	Brain connections	Frequency regions	*P* values
Cross correlation	RT-C	Delta (*δ*)	2.47 × 10^−4^
		Theta (*θ*)	1.46 × 10^−4^
		Alpha (*α*)	0.009
	RT-O	Delta (*δ*)	6.9 × 10^−5^
		Theta (*θ*)	2.7 × 10^−5^
		Alpha (*α*)	0.0029
	RT-F	Delta (*δ*)	5.01 × 10^−4^
		Theta (*θ*)	6.8 × 10^−5^
		Alpha (*α*)	0.0062
	LT-C	Delta (*δ*)	4.3 × 10^−5^
		Theta (*θ*)	3.8 × 10^−5^
		Alpha (*α*)	0.0192
	LT-O	Delta (*δ*)	8.5 × 10^−5^
		Theta (*θ*)	6.8 × 10^−5^
		Alpha (*α*)	0.0052
	LT-F	Delta (*δ*)	2.2 × 10^−4^
		Theta (*θ*)	5.4 × 10^−5^
		Alpha (*α*)	0.0091
	LT-RT	Delta (*δ*)	3.3 × 10^−4^
		Theta (*θ*)	6 × 10^−5^
		Alpha (*α*)	0.0253

Phase synchrony	RT-C	Delta (*δ*)	0.0067
		Theta (*θ*)	0.0403
		Alpha (*α*)	0.05
	RT-O	Delta (*δ*)	0.0041
		Alpha (*α*)	0.0271

Coherence	RT-C	Delta (*δ*)	0.0378
	RT-O	Delta (*δ*)	0.0378
		Alpha (*α*)	0.0192

**Table 2:** Total number of significant values in case of PCA and Average method.

Synchrony measure	Method	*P* < 0.01 (Total values)	*P* < 0.05 (Total values)
Cross correlation	PCA	26	35
	Average	22	30

Phase synchrony	PCA	8	11
	Average	2	8
